# A Subspace Approach to Sparse Sampling Based Data Gathering in Wireless Sensor Networks

**DOI:** 10.3390/s20040985

**Published:** 2020-02-12

**Authors:** Jingfei He, Xiaoyue Zhang, Yatong Zhou, Miriam Maibvisira

**Affiliations:** Tianjin Key Laboratory of Electronic Materials and Devices, School of Electronics and Information Engineering, Hebei University of Technology, 5340 Xiping Road, Beichen District, Tianjin 300401, China

**Keywords:** wireless sensor networks, data gathering, sparse sampling, subspace, data reconstruction

## Abstract

Data gathering is an essential concern in Wireless Sensor Networks (WSNs). This paper proposes an efficient data gathering method in clustered WSNs based on sparse sampling to reduce energy consumption and prolong the network lifetime. For data gathering scheme, we propose a method that can collect sparse sampled data in each time slot with a fixed percent of nodes remaining in sleep mode. For data reconstruction, a subspace approach is proposed to enforce an explicit low-rank constraint for data reconstruction from sparse sampled data. Subspace representing spatial distributions of the WSNs data can be estimated from previous reconstructed data. Incorporating total variation constraint, the proposed reconstruction method reconstructs current time slot data efficiently. The results of experiments indicate that the proposed method can reduce the energy consumption and prolong the network lifetime with satisfying recovery accuracy.

## 1. Introduction

Recently, wireless sensor networks (WSNs) have been applied in many fields, such as target tracking, medical care, and environmental monitoring. The component of a WSN includes a number of sensor nodes monitoring and collecting the physical environmental information and a sink aggregating the collected data. The data collection from each node to the sink, known as data gathering, is a significant research issue in WSNs.

In most real-world applications, the sensor nodes in WSNs are always limited by computational capacity and battery power. Lots of data gathering methods have been proposed to reduce energy consumptions in WSNs. These data gathering methods can be mainly classified into two categories: methods utilizing data compression techniques [1,2,3] and methods based on designing network protocols [4,5,6]. In the past decades, inspired by the emergence of compressed sensing (CS) [7] and matrix completion [8] theory in signal processing field, data gathering methods based on data compression techniques obtain more attention. It is important to note that the decrease in data transmission between nodes can effectively reduce energy consumption and prolong the network lifetime. Considering the redundant and highly correlated data sensed in neighboring sensors during consecutive times, data gathering methods taking advantage of data compression techniques have contributed to reducing the amount of data transmission and prolonging the network lifetime. Specifically, Compressive Data Gathering (CDG) was proposed [2] based on compressed sensing theory [7]. Instead of collecting each raw sensed data, the sink in CDG receives a weighted sum of all the readings from nodes. Compared with the traditional data gathering methods in WSNs, CDG can balance the energy consumption and prolong the lifetime of the network. Afterwards, many extensions to the CS based data gathering methods in WSNs have been developed [9,10,11] based on dense sensing matrix. To further reduce the amount of transmission data, methods utilizing sparse sensing matrix to random sample the raw sensed data in WSNs were also proposed [12,13,14]. More recently, as the rank of matrix is interpreted as a measure of second-order sparsity, matrix completion method [8] has attracted the attention of researchers, and many data gathering and reconstruction methods in WSNs based on matrix completion were proposed [3,15,16,17,18,19]. By distributing data collected from different nodes in different time slots into an environment matrix, the matrix completion based methods enforce the low-rank constraint on the matrix to take advantage of the spatiotemporal correlation. In particular, Spatio-Temporal Compressive Data Collection (STCDG) [3] was proposed to reduce the amount of traffic and improve the level of recovery accuracy by enforcing the low-rank constraint based on matrix factorization approach. Furthermore, methods utilizing joint low-rank and sparsity constraints were also proposed to further improve the data recovery accuracy [16,19]. Considering the inherent correlation among multi-attribute data, this method [19] extends the low-rank constraint based on matrix to tensor model to further exploit the correlation.

Although these methods enforcing low-rank constraint achieve better data recovery performances than the CS based methods, the requirement to form an environment matrix limits the real-time data gathering and reconstruction in WSNs. Besides, the sparse sampling strategy is adopted in matrix completion based methods, resulting in the existence of several nodes remaining in sleep mode. These inactive nodes will cause the failure of real-time data gathering. To achieve real-time data gathering with matrix completion, this paper proposes a sparse data gathering method based on clustered WSNs. Sparse sampled data can be collected in each time slot even with a fixed percent of nodes remaining in sleep mode. For data reconstruction, a subspace approach is proposed to enforce an explicit low-rank constraint. With subspace representing temporal distributions of the WSNs data estimated from previous reconstructed data, data collected in current time slot can be recovered efficiently. To guarantee data recovery performance even with low sampling ratio, we incorporate total variation constraint to further improve data recovery accuracy.

The rest of this paper is organized as follows. Section 2 reviews related works on matrix completion. Section 3 describes the proposed sparse data gathering method and the subspace approach for data reconstruction. The experimental results and analysis are presented in Section 4. Finally, Section 5 concludes this paper.

## 2. Matrix Completion in WSNs

### 2.1. Matrix Completion

Matrix completion problem was proposed to recover a data matrix from its partially sampled entries, and it has been proved that most low-rank matrices can be perfectly recovered from an incomplete set of entries [8]. Specifically, recovering an incomplete matrix $M\in R^{n\times m}$ can be cast as a rank minimization problem:

(1) $$\begin{array}{cc} \mathrm{minimize} & \mathrm{rank}\left(X\right)\\ \mathrm{subject}\;\mathrm{to} & X_{ij}=M_{ij}\;\;\left(i,j\right)\in \Pi , \end{array}$$

where X is the decision variable, Π the sampled subset of the complete set of entries [n]×[m] (Here and in the sequel, [n] denotes the list {1,…,n}), and $\mathrm{rank}\left(X\right)$ represents the rank of the matrix X. Essentially, the rank of matrix is treated as the measure of the second order sparsity. Therefore, the low-rank constraint can utilize the spatial and temporal correlation in data matrix. However, the optimization problem (1) is nonconvex and NP-hard. To address this issue, two popular approaches are utilized in numerous applications to enforce the low-rank constraint. One is the nuclear norm based approach, and the other is matrix factorization based approach. For the first one, since the nuclear norm is the best convex surrogate to the rank function over matrices with spectral norm less than or equal to one [20], the nuclear norm minimization is used as an alternative:

(2) $$\begin{array}{cc} \; & \mathrm{minimize}\;{∥X∥}_{*}\\ \; & \mathrm{subject}\;\mathrm{to}\;\;\;\;\;X_{ij}=M_{ij}\;\;\left(i,j\right)\in \Pi . \end{array}$$

Here, ${∥\cdot ∥}_{*}$ denotes the nuclear norm, which is a convex function and equal to the sum of the singular values of the matrix. Then, (2) can be rewritten as a regularized unconstrained problem, and singular value thresholding algorithm [21] can be used for the resulting problem. It is worth noting that computing Singular Value Decomposition (SVD) in each iteration is the main computational cost.

For the second one, the incomplete matrix M with rank *r* can be expressed as the product of two matrices LR, where $L\in R^{n\times r}$, $R\in R^{r\times m}$. The matrix factorization is not unique, the factorization where the matrices L and R have Frobenius norm as small as possible can be searched. Then (1) can be cast as:

(3) $$\begin{array}{cc} \; & \mathrm{minimize}\;\frac{1}{2}\left({∥L∥}_{F}^{2}+{∥R∥}_{F}^{2}\right)\\ \; & \mathrm{subject}\;\mathrm{to}\;\;\;\;\;{\left(\mathrm{LR}\right)}_{ij}=M_{ij}\;\;\left(i,j\right)\in \Pi , \end{array}$$

The matrix factorization based approach has gained great popularity due to fewer requirements for storage capacity and computational overhead. It is worth noting that the problem (3) is a nonconvex quadratic program and can be solved by standard nonlinear optimization algorithms, such as alternating minimization method [22] and gradient descent method.

### 2.2. Matrix Completion Based Method in WSNs

Inspired by the great success of matrix completion, many data gathering and reconstruction methods in WSNs were proposed based on matrix completion. Considering a WSN consisting of *n* sensor nodes and one sink with symbol $N_{1},N_{2},\cdots ,N_{n}$ used to represent sensor nodes. These sensor nodes sense the environment and transmit readings to the sink once every τ time. Therefore, an environment matrix (EM) M organized by n×m readings can be obtained during mτ time (i.e., *m* time slots):

(4) $$M=\left[\begin{array}{ccc} f\left(N_{1},T_{1}\right) & \cdots & f\left(N_{1},T_{m}\right)\\ \vdots & \ddots & \vdots \\ f\left(N_{n},T_{1}\right) & \cdots & f\left(N_{n},T_{m}\right) \end{array}\right]\in R^{n\times m},$$

where $f\left(N_{i},T_{j}\right)$ denotes the reading collected from node $N_{i}$ in the *j*th time slot. In most matrix completion based methods, partially sampled readings sensed during *m* time slots are transmitted to the sink together. Since readings generated by the nodes in a certain area during consecutive times are redundant and highly correlated, the EM M has approximately low-rank structure. Then, the low-rank constraint can be enforced to exploit the correlation to reconstruct the unsampled readings. Methods based on matrix completion have achieved great success. However, the requirement to form an EM limits the real-time data gathering and reconstruction in WSNs.

## 3. The Proposed Method

In matrix completion based methods in WSNs, sparse sampling is adopted in data gathering to reduce the amount of transmission data and energy consumption. Typically, the sparse sampled readings sensed during a fixed number of time slots are transmitted to the sink together in the last time slot, which limits real-time data gathering and reconstruction. To implement real-time data gathering in WSNs, the sparse sampled data should be collected and transmitted to the sink in every time slot. However, since only partial sensor nodes wake up and collect data while other nodes remaining in sleep mode, the traditional data gathering methods fail due to the existence of inactive nodes. If the successful aggregation of sparse sampled data is guaranteed at the expense of all nodes waking up to transmit data, the goal of reducing energy consumption through sparse sampling can not be achieved. Moreover, for data reconstruction in real-time case, only data in current time slot need to be reconstructed. In this paper, a sparse data gathering method based on clustered WSNs is proposed to implement real-time data gathering, and a subspace approach based method is proposed to reconstruct data in current time slot by enforcing an explicit low-rank constraint.

### 3.1. The Sparse Sampling Data Gathering Scheme

**Figure 1:**
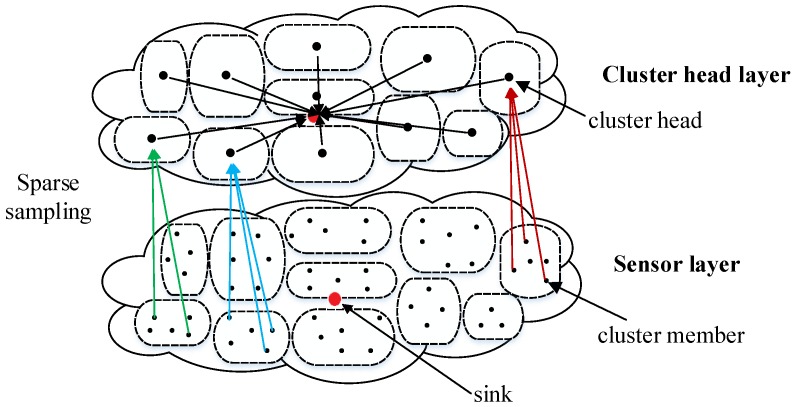
The sparse sampling data gathering scheme in wireless sensor networks (WSNs).

To ensure the success of sparse sampled data gathering without waking up all nodes, a sparse sampling data gathering scheme is proposed in clustered WSNs. In clustered WSNs, all nodes are divided into multiple clusters, and each cluster contains one cluster head (CH) and a number of cluster members (CMs). The cluster head is selected for each round to communicate with the sink, and the cluster members only communicate with CHs in their clusters. The proposed sparse sampling data gathering scheme based on clustered WSNs is shown in Figure 1. For clarity, nodes are divided into two layers: sensor layer containing CMs in each cluster and cluster head layer containing all CHs. Sparse sampling is adopted for CMs in each cluster, that is, only partial CMs wake up and transmit the sensed data to the CH. The CHs transmit the received data and its own sensed data to the sink. It is worth noting that several CHs which locate far from sink node can communicate with sink using multi-hop transmission. For simplicity, only direct communication between CHs and the sink is illustrated in Figure 1.

The proposed sparse sampling data gathering scheme can combine with many existing clustering algorithms. In this paper, the well known distributed energy-efficient clustering (DEEC) scheme [4] is selected as the clustering algorithm. In DEEC, cluster heads are selected according to a probability based on the ratio between the residual energy of each node and the average energy of the network. Because of adapting the rotating epoch of each node to its energy, the nodes with high initial and residual energy have a higher probability of becoming CHs than those with low energy. Specifically, the proposed sampling data gathering scheme using DEEC can be divided into rounds, and each round contains two phases as follows.

In the first phase, clusters are formed and the sampling ratio of each node is determined. Specifically, each node decides whether to become a CH based on its own probability threshold, which is related to the residual energy and estimated average energy of networks at current round. After the CHs are selected, the other nodes, cluster members, determine the dependent cluster according to the signal strength of the received information, and notify the corresponding CH to complete the establishment of the clusters. Then the sink broadcasts a fixed sampling ratio ρ to all sensor nodes in the network. Note that the CHs are always awake to ensure the successful aggregation of data. As a result, the sampling ratio for CHs can be set to 1. For the CMs, the sampling ratio is ρCH=ρn−nCHρn−nCHn−nCHn−nCH, where *n* denotes the number of nodes still alive and $n_{CH}$ the number of CHs. $n-n_{CH}$ represents the number of CMs.

In the second phase, sparse sampling and data gathering are held. Specifically, each node in CMs generates a random number between 0 and 1. If the number is less than the sampling ratio $\rho_{CH}$ then the node senses the environment and transmits readings to the corresponding CH, otherwise the node remains in sleep mode in current time slot. After receiving data from CMs in its cluster, the CH transmits the received data and its own sensed data to the sink. The network will start a new round after a fixed number of time slots.

Instead of using all nodes to collect the information, the proposed sampling data gathering scheme only wakes partial nodes up in each time slot, which greatly reduces the energy consumption and prolongs the lifetime of the network compared to original clustering algorithm.

### 3.2. The Sliding Window Model

**Figure 2:**
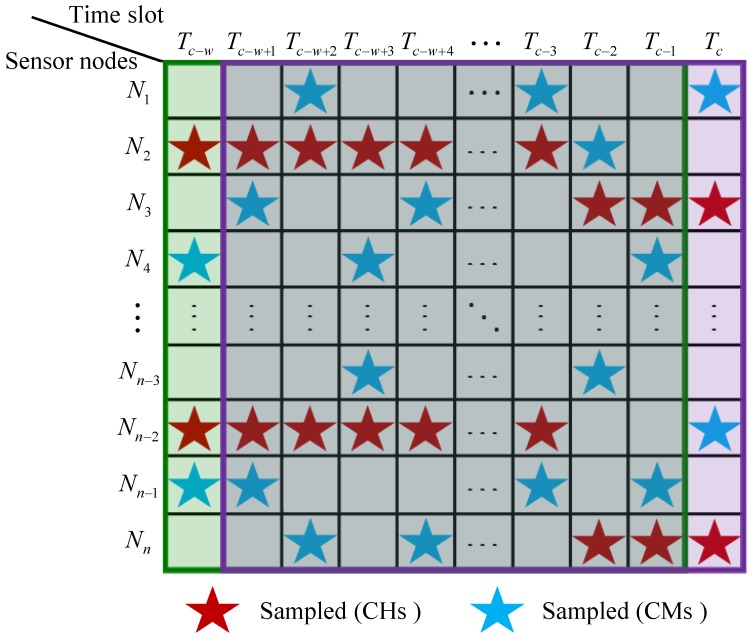
Sampled data using sliding window model.

In real-time data gathering with sparse sampling, data from CHs and partial CMs are collected in the sink for current time slot. We use $T_{c}$ to represent the current time slot, and let $T_{c-j}$ denote the last (j+1)th time slot. Figure 2 shows the data sampled from *n* sensor nodes between time slots $T_{c-w}$ to $T_{c}$. To implement the real-time data reconstruction, the sliding window model is utilized by introducing the reconstructed data in previous time slots. As illustrated in Figure 2, two adjacent windows are shown with the width fixed to *w* time slots. The first window, the previous window of the current window, contains time slots from $T_{c-w}$ to $T_{c-1}$, and the second window, the current window, contains time slots from $T_{c-w+1}$ to $T_{c}$. Data in each window can form an environment matrix, and the window slides forward a time slot each time. That is, the oldest time slot will be deleted, while the current time slot enters the window. Meanwhile, the environment matrix is updated accordingly.

For example, to reconstruct data $x_{c}$ in current time slot $T_{c}$, the previous window containing time slots $T_{c-w}$ to $T_{c-1}$ slides forward a time slot. Then the current window including data from $T_{c-w+1}$ to $T_{c}$ is obtained. Divide the correspondingly current EM $\left[M_{p}\;x_{c}\right]$ into two parts: the previous reconstruction data $M_{p}$ (data in time slots $T_{c-w+1}$ to $T_{c-1}$) and the current data $x_{c}$ (collected in current time slot). Since each column in $M_{p}$ has been reconstructed in the corresponding time slot, the proposed subspace approach can enforce an explicit low-rank constraint to reconstruct the current data $x_{c}$ in real time.

It is worth noting that the sink needs the indexes of the sparse sampled data to reconstruct the current data. To avoid overhead incurred by index information, the preset random seed can be used. At the beginning of each round, each CM generates a random seed as a pseudo-random number generator and sends it to the CHs and sink. The generated random number determines whether the CM wakes up and collects data. With all the information of random seeds, the sink can obtain the indexes of the sparse sampled data without extra overhead.

### 3.3. The Subspace Approach for Reconstruction

With the proposed sparse sampling data gathering scheme, the sink can obtain the current sparse sampled data $d=\Omega (x_{c})$, where $\Omega :R^{n\times 1}\to R^{s\times 1}$ is the sparse sampling operator to obtain the partially known data from the current data. According to the sliding window model, the current EM can be expressed as $\left[M_{p}\;x_{c}\right]$. Readings in EM are collected from nodes in a certain area during a consecutive time, then EM has approximately low-rank structure. Using the matrix factorization based approach, we have $[M_{p}\;x_{c}]=\mathrm{LR}$. Here, $L\in R^{n\times r}$ and $R\in R^{r\times w}$ can be regarded as the subspace representing the spatial distributions and the temporal distributions of the WSNs data, respectively. With R rewritten as $\left[R_{p}\;r_{c}\right]$, where $R_{p}\in R^{r\times (w-1)}$ and $r_{c}\in R^{r\times 1}$, we have $\left[M_{p}\;x_{c}\right]=L\left[R_{p}\;r_{c}\right]=\left[LR_{p}\;Lr_{c}\right]$. That is, $M_{p}=LR_{p}$ and $x_{c}=Lr_{c}$. Since the subspace L representing the spatial distributions can be estimated from previous reconstructed data $M_{p}$, the current data can be reconstructed by

(5) $$\begin{array}{cc} {\hat{r}}_{c} & =\arg\min_{r_{c}}\;{∥d-\Omega \left(Lr_{c}\right)∥}_{2}^{2}\\ {\hat{x}}_{c} & =Lr_{c}. \end{array}$$

To further improve the recovery accuracy, the total variation constraint $∥\nabla_{x}\left(\left[M_{p}\;x_{c}\right]\right){∥}_{1}$ can be jointly utilized to enforce the temporal stability, where $\nabla_{x}$ represents the horizontal finite difference operator. It is worth noting that the vertical finite difference operator is not used. Since adjacent nodes in the matrix may not adjacent in space, constraining the vertical finite difference can not utilize the spatial stability. Besides, $M_{p}$ is known by previous reconstruction, then the constraint $∥\nabla_{x}\left(\left[M_{p}\;x_{c}\right]\right){∥}_{1}$ can be simplified as $∥\nabla_{x}\left(\left[m_{p}\;x_{c}\right]\right){∥}_{1}$, where $m_{p}$ is the last column of $M_{p}$. By jointly enforcing the constraint and introducing a quadratic penalty term, (5) can be converted into a corresponding unconstrained formulation as:

(6) $$\begin{array}{cc} \; & {\hat{r}}_{c}=\arg\min_{r_{c}}{∥d-\Omega (Lr_{c})∥}_{2}^{2}+\lambda {∥\nabla_{x}\left(\left[m_{p}\;Lr_{c}\right]\right)∥}_{1}\\ \; & {\hat{x}}_{c}=Lr_{c}. \end{array}$$

where λ denotes the regularization parameter. The proposed subspace approach incorporates both the explicit low-rank constraint and the temporal stability constraint in a single formulation (i.e., (6)). To solve the optimization problem in (6), an efficient algorithm based on alternating direction method of multipliers (ADMM) [23,24] is developed. First, using variable splitting, we can convert (6) into the following equivalent constrained optimization problem:

(7) $$\begin{array}{cc} \; & \left\{{\hat{r}}_{c},y\right\}=\arg\min_{{\hat{r}}_{c},y}∥d-\Omega \left(Lr_{c}\right){∥}_{2}^{2}+\lambda {∥y∥}_{1}\\ \; & \;s.t.\;\;\;y=\nabla_{x}\left(\left[m_{p}\;Lr_{c}\right]\right). \end{array}$$

Second, the augmented Lagrangian function for (7) can be obtained:

(8) $$L\left(r_{c},y,a\right)=∥d-\Omega \left(Lr_{c}\right){∥}_{2}^{2}+\lambda {∥y∥}_{1}+\langle a,y-\nabla_{x}\left(\left[m_{p}\;Lr_{c}\right]\right)\rangle +\frac{\alpha }{2}{∥y-\nabla_{x}\left(\left[m_{p}\;Lr_{c}\right]\right)∥}_{2}^{2},$$

where a is the Lagrangian multiplier, and α>0 is the penalty parameters. Third, (8) can be minimized alternatively as following:

(9) $$\begin{array}{cc} \; & r_{c}^{k+1}=\arg\min_{r_{c}}L\left(r_{c},y^{k},a^{k}\right)=\arg\min_{r_{c}}{∥d-\Omega \left(Lr_{c}\right)∥}_{2}^{2}+\frac{\alpha }{2}{∥y^{k}-\nabla_{x}\left(\left[m_{p}\;Lr_{c}\right]\right)+\frac{a^{k}}{\alpha }∥}_{2}^{2} \end{array}$$

(10) $$\begin{array}{cc} \; & y^{k+1}=\arg\min_{y}L\left(r_{c}^{k+1},y,a^{k}\right)=\arg\min_{y}{∥y∥}_{1}+\frac{\alpha }{2\lambda }{∥y-\left(\nabla_{x}\left(\left[m_{p}\;Lr_{c}^{k+1}\right]\right)-\frac{a^{k}}{\alpha }\right)∥}_{2}^{2} \end{array}$$

(11) $$\begin{array}{cc} \; & a^{k+1}=a^{k}+\alpha \left(y^{k+1}-\nabla_{x}\left(\left[m_{p}\;Lr_{c}^{k+1}\right]\right)\right). \end{array}$$

To solve the subproblem in (9), which is a quadratic optimization problem, the preconditioned conjugate gradient (PCG) algorithm is applied in this paper. To solve the subproblem in (10), the well-known soft-thresholding formula [25] can be utilized. Then we have

(12) $$y^{k+1}=S\left(\nabla_{x}\left(\left[m_{p}\;Lr_{c}^{k+1}\right]\right)-\frac{a^{k}}{\alpha },\frac{\lambda }{\alpha }\right),$$

where $S{\left(q,\tau \right)}_{i}:=\mathrm{sign}\left(q_{i}\right)\max(|q_{i}\left|-\tau ,0\right)$ is a soft-thresholding operator for each element $q_{i}$ in q. The procedures of the reconstruction algorithm based on ADMM for solving (6) can be summarized in Table 1. In practical implementation, we initialize $r_{c}^{0}$, $y^{0}$, and $a^{0}$ with zeros vectors. Since (6) is a convex optimization problem, the ADMM based method is guaranteed to have global convergence from any initializations [24]. The stopping criteria for the algorithm are $∥r_{c}^{k}-r_{c}^{k-1}{∥}_{2}/{∥r_{c}^{k-1}∥}_{2}\leq \epsilon$ and $k>K_{max}$, where ϵ and $K_{max}$ are the predefined tolerance parameter and the maximum number of iterations, respectively. The algorithm is terminated until each criterion is satisfied.

## 4. Experiments and Analysis

In this section, the experimental environments are established to verify the effectiveness of the proposed method. For the reconstruction experiments, the performance of the subspace based reconstruction method was compared with local interpolation method K-Nearest Neighbors (KNN) [26], the CS based method [13], Seq-Prog-CS method [10], and joint CS and matrix completion method (CSMC) [16], and the real dataset collected from GreenOrbs was adopted. For the energy consumption experiments, the proposed sparse sampling data gathering scheme using DEEC was compared with the original DEEC to verify the benefit of the proposed method in reducing the energy consumption. All experiments were conducted using MATLAB R2017a on a computer with 1.8GHz Intel core i7-8550U CPU and 8GB RAM.

### 4.1. Data Reconstruction Performance

To verify the effectiveness of the subspace approach for reconstruction, a small real dataset collected from GreenOrbs [27] was selected as the ground truth. The readings in the small real dataset were collected from 94 nodes in 124 time slots for three attributes: temperature, humidity, and light. Then we verified the effectiveness of the proposed method from two aspects. One is the recovery of data in current time slot. Another one is the long-term recovery of data in consecutive time slots.

For the first aspect, the 50th time slot in the dataset was selected as the current time slot, and the width of sliding window was set to w=41. $x_{c}\in R^{n\times 1}$ denote the data in current time slot, and $M_{p}\in R^{n\times 40}$ denotes the previous reconstructed data. Here, n=94 is the number of nodes. With predefined sampling ratio ρ, only partial readings $d\in R^{\left\lfloor n\rho \right\rfloor \times 1}$ can be sampled using the proposed sparse sampling data gathering scheme. Mathematically, the sampling procedure can be expressed as $d=\Omega (x_{c})$. The proposed method and compared methods were used to reconstruct ${\hat{x}}_{c}$ from d. The Normalized Mean Absolute Error (NMAE) was adopted to measure the recovery performance and defined as:

(13) $$NMAE=\frac{\sum_{i\in \Pi }\left|x_{c_{i}}-{\hat{x}}_{c_{i}}\right|}{\sum_{i\in \Pi }\left|x_{c_{i}}\right|}.$$

Here, ${\hat{x}}_{c}$ is the recovered data, and Π represents the unsampled subset of the complete set of entries [n]×1. That is, we only calculated the recovery accuracy of the unsampled data.

In the simulation, the spatial distributions L need to be estimated for the proposed subspace approach, which need $M_{p}$ and the rank *r*. For simplicity, the historical data collected from 10th to 49th time slots were used as $M_{p}$, and the rank *r* was set to 15 according to singular values of $M_{p}$. Besides, to make the comparison more convincing, the same historical data were also used in CSMC and Seq-Prog-CS method. The sampling ratio ρ was set as [0.4,0.3,0.2,0.1,0.07,0.04,0.01], and the sampling operator Ω was generated with a uniform random sampling pattern. Each experiment was repeated 100 times to calculate the mean NMAE value for each method. For each experiment, the same sampling operator and sampled data were used for all the methods.

**Figure 3:**
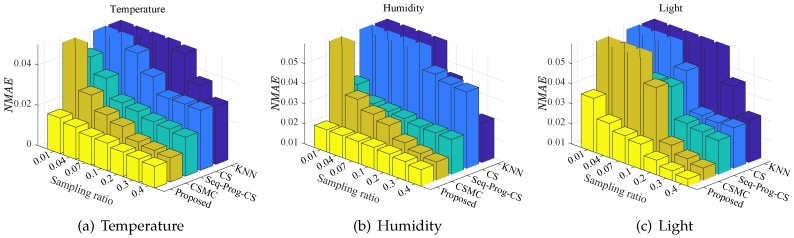
The recovery performance of KNN, CS, Seq-Prog-CS, CSMC, and the proposed method for three attributes in GreenOrbs: (**a**) temperature, (**b**) humidity, and (**c**) light.

Figure 3 shows the recovery performance of methods for the temperature, humidity, and light data in GreenOrbs, respectively.

As shown in Figure 3, the proposed method achieves the lowest NMAE with each sampling ratio for all attributes. With the decrease of sampling ratio, the performance of other three methods degrades dramatically while the proposed method keeps satisfying performance. Even with the sampling ratio as low as 0.01, the proposed method obtains NMAE =0.018,0.021,0.037 for temperature, humidity, and light data, respectively.

**Figure 4:**
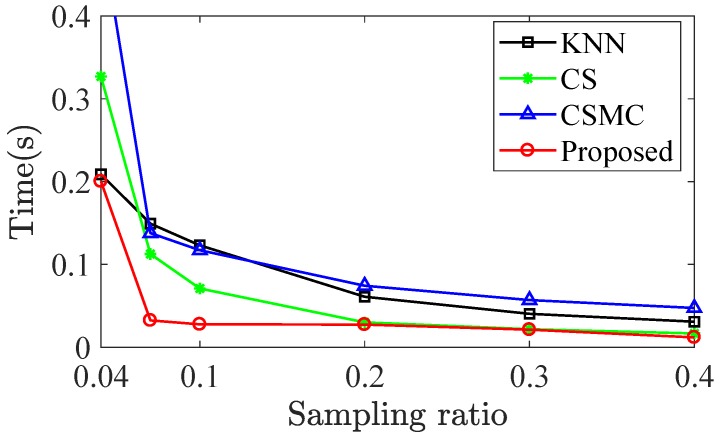
Running time over sampling ratio for temperature data in GreenOrbs.

Figure 4 shows the running time performance of methods for temperature data in GreenOrbs. The average running time of KNN, CS, CSMC, and the proposed method is graphically represented. As shown in Figure 4, the proposed method achieves the lowest running time. The reason is that the proposed method avoids the singular value decomposition (SVD) in (6). For traditional matrix completion methods, the main computational complexity is from the SVD of EM for each iteration, and exact SVD of a matrix with size n×m has time complexity $O(\min\left\{nm^{2},n^{2}m\right\})$. In the proposed subspace approach, the SVD is used to obtain the subspace L from previous reconstructed data $M_{p}$. The calculation only needs to be run once and can be done between two time slots. Then the computational complexity in each iteration to solve (6) is negligible.

**Figure 5:**
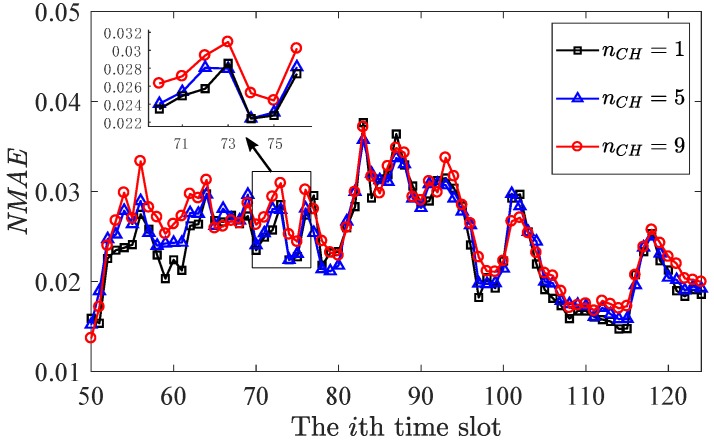
Long-term recovery of the proposed method for the temperature data in GreenOrbs.

The historical data were used as $M_{p}$ in the above simulation, while $M_{p}$ should be the previous reconstructed data in the practical implementation. It is necessary to verify the effectiveness of the proposed method in long-term reconstruction, which updates $M_{p}$ with previous reconstruction and estimates spatial distribution L for next time slot recovery. Then for the second aspect, the long-term reconstruction, data collected from the 50th time slot to the 124th time slot in the dataset were selected. Set the number of cluster head $n_{CH}$ as [1,5,9] and the sampling ratio ρ=0.8. Other settings were set as the previous simulation. Figure 5 shows the long-term recovery performance of the proposed method for the temperature data in GreenOrbs.

Despite the reduction in the recovery accuracy of the proposed method for long-term reconstruction, the overall reconstruction accuracy is still satisfying. The average NMAE is [0.024,0.0245,0.0254] for the proposed method with [1,5,9] cluster heads, respectively. It is obvious that less number of cluster head leads to lower NMAE. With a fixed number of CH, only $\left\lfloor n\rho \right\rfloor -n_{CH}$ sampling nodes are randomly selected while $n_{CH}$ nodes are fixed. As a result, if $n_{CH}=0$, all sampling nodes are randomly selected, which can achieve better recovery.

### 4.2. The Energy Consumption Performance

To further verify the benefit of the proposed method in reducing the energy consumption, we established the experimental environments for the proposed sparse sampling data gathering scheme using DEEC as the clustering algorithm.

In the simulation, a two-level heterogeneous network containing advanced nodes and normal nodes was utilized. The initial energy of normal node was $E_{0}$, and the initial energy of advance node was $aE_{0}$. There were 100 sensors (80 advanced nodes and 20 normal nodes) randomly distributed in a 100m×100m area and one sink located in the center of the area.

The parameters used in simulations are shown in Table 2. The transmission process adopted free space model and the multipath fading model for distance d≤87m and d>87m, respectively. There were 100 times data gathering in one round, and the cluster heads were reselected at the beginning of each round. The number of cluster head was set to 10 for both methods, and the sampling ratio was set as [0.2,0.4,0.6] for the proposed method.

**Figure 6:**
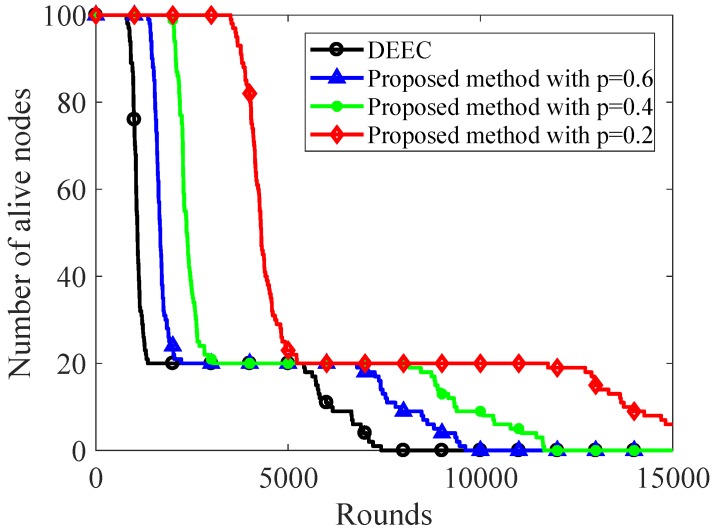
Number of alive nodes over rounds.

**Figure 7:**
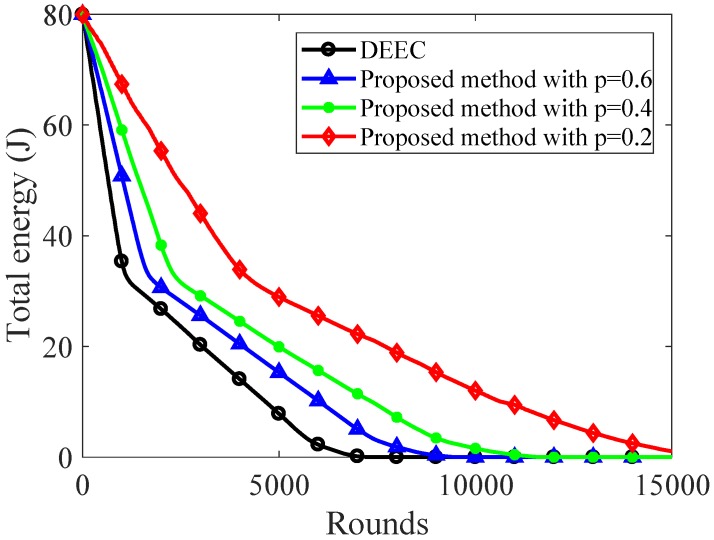
Total energy over rounds.

Figure 6 and Figure 7 show the energy consumption of original DEEC and the proposed sparse sampling data gathering scheme using DEEC. As shown in Figure 6, the nodes start to die in 798th round for original DEEC, while the proposed method keeps all nodes alive until the 1356th, 1941th, 3504th round with ρ=0.6, ρ=0.4, ρ=0.2, respectively.

To keep 50% nodes alive, the original DEEC can only carry out 1081 rounds. The proposed method with ρ=0.2 can carry out 4300 rounds, which is about 4 times than that of original DEEC. As the sampling ratio decreases, more network nodes can keep alive after a fixed number of rounds. As shown in Figure 7, the total energy of network decreases slowly using the proposed sparse sampling data gathering scheme. When the sampling ratios are 0.2, 0.4, and 0.6, the total energy of the proposed method is 33.9J, 24.55J, and 20.46J, respectively, while the original DEEC only had 14.1J at the 4000th round. To keep 20% total energy, the original DEEC can only carry out 3692 rounds, while the proposed method with ρ=0.2 can carry out 8829 rounds. It is obvious that the proposed method can reduce the energy consumption significantly compared with the original DEEC.

## 5. Conclusions

This paper proposes an efficient sparse sampling data gathering method in clustered WSNs including a sparse sampling data gathering scheme and a subspace based reconstruction algorithm. The proposed sparse sampling data gathering scheme not only uses the sparse sampling strategy to reduce the amount of transmission data and the energy consumption, but also ensures the success of real-time sparse sampled data gathering in clustered WSNs. Then the previous reconstructed data are introduced with the sliding window model and used to estimate the subspace representing spatial distributions of the WSNs data. In this way, the proposed subspace based reconstruction approach can enforce an explicit low-rank constraint for data reconstruction from sparse sampled data. Besides, the total variation constraint is joint utilized to further improve the recovery accuracy. The experimental results show that the proposed method outperforms the state-of-the-art methods in data recovery and reduces the energy consumption of network efficiently.

## Figures and Tables

**Table 1:** The reconstruction algorithm to solve the optimization problem in (6).

**Input:**
Initialized $r_{c}^{0}$,$y^{0}$,and $a^{0}$ with zeros vectors;
The subspace L representing the spatial distributions;
The regularization parameter λ and the penalty parameter α;
The measurements d, the sampling operation Ω, and the iteration number k=0;
**do**
1)Iteration number k=k+1;
2)Update $r_{c}^{k}$ by solving (9);
3)Update $y^{k}$ by solving (12);
4)Update $a^{k}$ by solving (11);
**while**$\frac{{∥r_{c}^{k}-r_{c}^{k-1}∥}_{2}}{{∥r_{c}^{k-1}∥}_{2}}>\varepsilon$**and**$k\leq K_{max}$;
**Output:** ${\hat{x}}_{c}=Lr_{c}^{k}$

**Table 2:** Network energy consumption model.

Description	Value
*a*	4
Initial energy of normal node $E_{0}$	0.5J
Energy for transmit per bit	50nJ/bit
Energy for receiving per bit	50nJ/bit
Amplifier energy for free space model	10pJ/bit/$m^{2}$
Amplifier energy for multipath fading model	0.0013pJ/bit/$m^{4}$
